# Automatic Analysis of Archimedes’ Spiral for Characterization of Genetic Essential Tremor Based on Shannon’s Entropy and Fractal Dimension

**DOI:** 10.3390/e20070531

**Published:** 2018-07-16

**Authors:** Karmele Lopez-de-Ipina, Jordi Solé-Casals, Marcos Faúndez-Zanuy, Pilar M. Calvo, Enric Sesa, Josep Roure, Unai Martinez-de-Lizarduy, Blanca Beitia, Elsa Fernández, Jon Iradi, Joseba Garcia-Melero, Alberto Bergareche

**Affiliations:** 1 Systems Engineering and Automation Department, EleKin Research Group, University of the Basque Country UPV/EHU, 20018 Donostia, Spain; 2 Data and Signal Processing Research Group, University of Vic—Central University of Catalonia, 08500 Barcelona, Spain; 3 Escola Superior Politècnica Tecnocampus, Universitat Pompeu Fabra, 08002 Barcelona, Spain; 4 Department of Electronic Technology, EleKin Research Group, University of the Basque Country UPV/EHU, 20018 Donostia, Spain; 5 Department of Mathematics, EleKin Research Group, University of the Basque Country UPV/EHU, 1006 Gasteiz, Spain; 6 Department of Enterprises Organization, EleKin Research Group, University of the Basque Country UPV/EHU, 20018 Donostia, Spain; 7 Department of Mechanical Engineering, EleKin Research Group, University of the Basque Country UPV/EHU, 1006 Gasteiz, Spain; 8 Department of Neuroscience, BioDonostia Health Institute, 20014 Donostia, Spain

**Keywords:** essential tremor, automatic analysis of drawing, spiral of Archimedes, entropy, fractal dimension, automatic selection of features

## Abstract

Among neural disorders related to movement, essential tremor has the highest prevalence; in fact, it is twenty times more common than Parkinson’s disease. The drawing of the Archimedes’ spiral is the gold standard test to distinguish between both pathologies. The aim of this paper is to select non-linear biomarkers based on the analysis of digital drawings. It belongs to a larger cross study for early diagnosis of essential tremor that also includes genetic information. The proposed automatic analysis system consists in a hybrid solution: Machine Learning paradigms and automatic selection of features based on statistical tests using medical criteria. Moreover, the selected biomarkers comprise not only commonly used linear features (static and dynamic), but also other non-linear ones: Shannon entropy and Fractal Dimension. The results are hopeful, and the developed tool can easily be adapted to users; and taking into account social and economic points of view, it could be very helpful in real complex environments.

## 1. Introduction

Essential tremor (ET) is a worldwide disease that is twenty times more common than Parkinson’s disease (PD) [1]. In the western world, the prevalence of this motor disorder is about 0.3% to 4.0%. Its incidence is 23.7 per 100,000 individuals per year, and both men and women of 40 years old are affected more or less in the same proportion; there are no differences with regard to gender. Studies point out that 50–70% of ET cases are estimated to be genetic [1]. ET is a rhythmic tremor (4 to 12 Hz) which only manifests when the muscle is exerting, and the tremor’s amplitude varies as the patient becomes older. ET is characterized by both kinetic and postural tremor, and most of the times it affects the hands. Almost every other tremor occurs combined with hand tremor [2].

The clinical diagnosis of the first manifestations of the disease is crucial to palliate and manage the symptoms, since they produce problems in the performance in daily activities. In recent years we have witnessed notorious advances in early diagnosis techniques and in the creation of usable clinical markers. The differential diagnosis between PD and ET is clinical. When the diagnosis is doubtful, a SPECT-DAT scan can be performed, which consists in evaluating the striatal uptake of 123I-2b-carbomethoxy-3b-(4-iodophenyl)-*N*-(3-fluoropropyl)-nortropane using a SPECT (Single Photon Emission Computed Tomography), a test that is extremely expensive, and therefore not easily accessible in habitual clinical practice [3]. In spite of the usefulness of these biomarkers, the expenses and requirements of the needed technology make it unfeasible to use these tests with every patient that has motor conditions. In this context, intelligent techniques that are non-invasive for the diagnosis can be very useful tools for early diagnosis of neurodegenerative disorders. Staff who are non-technical in the environment of the patient could be able to use these techniques and other biosignals without interfering with the patient’s daily life, as analysis of speech, analysis of handwriting or analysis of drawings are not considered stressful by most patients. Furthermore, the cost of these techniques is low and their requirements regarding infrastructure or medical equipment are not extensive, whereas they provide information in an easy, quickly, and inexpensive way [4,5,6,7,8]. Medical staff commonly use handwriting tasks for diagnosing essential tremor. Nowadays, Archimedes’ spiral is the gold standard test in clinical diagnosis [9].

The analysis of handwriting has traditionally been performed off-line, as only the strokes on the paper were available for the analysis. Nowadays, new devices, such as digitizing tablets and pens (with ink or without), can collect both written data and their temporal information (on-line handwriting analysis). New digitizing tablets gather not only the x and y coordinates of the movement of the device of writing, but they also gather other data, for example: the pressure on the surface of the writing device, the azimuth (angle between the pen and the horizontal plane), the altitude (angle between the pen and the vertical axis), or even the in-air movement when there is no contact between the pen and the writing surface. All these capabilities make it. possible to analyze both static and dynamic features [10,11].

In the field of medicine, the analysis of handwriting has found to be useful to make a diagnosis and track neurodegenerative diseases. For example, the degradation of handwriting skills and Alzheimer’s Disease (AD) seem to be closely related [12,13], and several handwriting-related aspects can be used as indicators for diagnosing it [12], or could differentiate mild AD from mild cognitive impairment [12,13]. Handwriting analysis has also been shown to be helpful to study how substances such as alcohol [14], marijuana [15], or caffeine [16] affect patients. Psychology has benefited, as well, from the analysis of handwriting thanks to new devices of acquisition. For example, Rosenblum et al. [17] report the relation between the ability of the writers and the duration of the in-air trajectories of the handwriting. The progressive degree of impairment can be noticed by visually inspecting the pen-down image, when the drawing is more disorganized, and the effect in three dimensions is only noticed for mild cases of ET and PD patients. There are currently references that analyze the detection of ET by means of digital platforms. In [18], new numerical techniques are proposed in order to evaluate them with regard to the agreement with visual rating and reproducibility. For example, [19] analyzes the increase in the variability of the drawing of the spiral in patients with functional (psychogenic) tremor. Louise presents an interesting study over four cohorts of ET [20]. On the other hand, recent references [21,22,23,24] present advances in PD analysis by means of digital devices as well. The visual information from the in-air trajectories of the drawings of unhealthy people also shows disorganization and a progressive impairment when those people try to plan their drawings. It is worth noticing that if pen-up images are compared between unhealthy individuals and subjects in the control group, it also reveals important differences. In addition to a longer in-air time, there are more hand-movements before they put the pen back on the surface. Therefore, these measures of graph-motor type can be useful when applied to the analysis of writing and drawing in order to analyze the precise nature and progression of the writing and drawing disorders related to neurodegenerative diseases.

The work presented in this paper is part of a larger cross study aimed at diagnosing and characterizing essential tremor. This research is conducted at the Biodonostia Health Institute, and it analyzes families with identified genetic loci [25,26]. Nevertheless, the study presented in the manuscript is aimed at the characterization of essential tremor, and its objectives are focused on establishing the limits related to the natural variability of ET and the ability to discriminate ET from physiological tremor. Therefore, the subject of study is not the ability to make a differential diagnosis with PD. Regarding the gathering of non-linear biomarkers from handwriting and drawings, Archimedes’ spiral is the test to be explored. This test is also able to detect other diseases such as stress, which could appear in control individuals. In the next Sections, we will analyze not only commonly used dynamic and static linear features, but other non-linear ones as well, i.e., fractal dimension and entropy. Potential biomarkers will be identified by means of several automatic classification techniques. Lastly, the quality of the selected features will be automatically analyzed by using Machine Learning paradigms.

## 2. Materials

### 2.1. System of Acquisition

**Figure 1:**
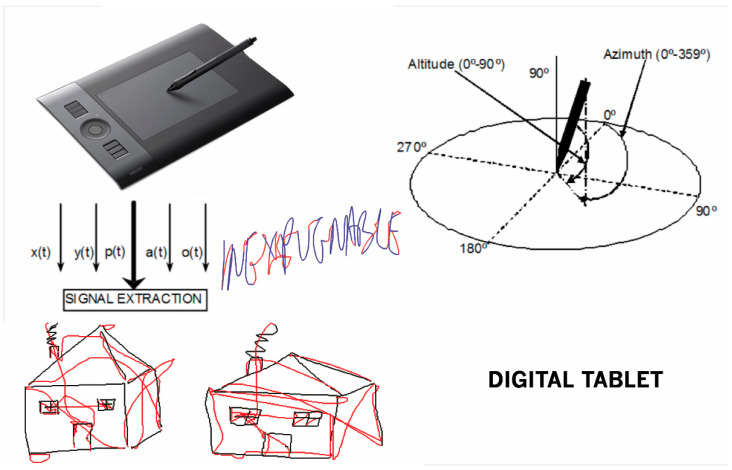
Information from the digitizing tablet.

The acquisition is performed with an Intuos Wacom 4 digitizing tablet. Figure 1 shows the information, which is captured by the USB pen. The tablet gathers 200 samples/second: spatial coordinates (x, y), the applied pressure of the pen, and a pair of angles, which are the azimuth (angle between the pen and the horizontal plane), and the altitude (angle between the pen and the vertical axis) [10,11]. Collecting only these dynamic features, afterwards, more information can be inferred. For example, velocity, acceleration, tangential acceleration, centripetal acceleration, angle of the instantaneous trajectory, instantaneous displacement, radius of curvature, etc. [26]. In Figure 1, there is a modern digitizing tablet, and the graphic representation of the angles of the azimuth and altitude. It also represents the capture of a handwritten text (the Spanish word “biodegradable”—i.e., biodegradable in English but with a different pronunciation), and two captures of the drawing of a house. Please note that every capture comprises both on-surface and in-air trajectories.

### 2.2. Database of Individuals

**Figure 2:**
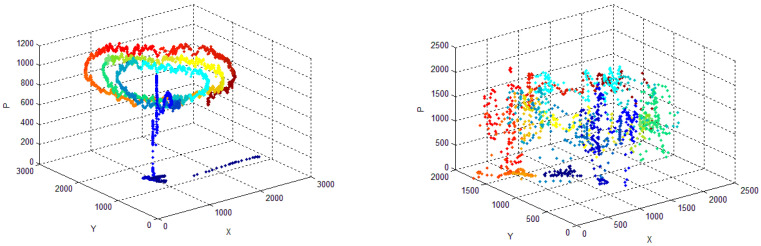
Example of (X, Y) and pressure (P) of the Archimedes’ spiral carried out by two individuals with ET: low level (**left image**); and high level (**right image**). Time evolution is shown in different colours (beginning in blue and ending in red).

This paper is based on a database named BIODARW, which contains samples from both hands of 50 people: 21 control individuals (CR) and 29 ET patients with identified genetic loci. The dataset belongs to a larger research study on the diagnosis of ET conducted by the Biodonostia Health Institute aimed at characterizing this impairment. The participants are families with identified genetics loci. Subjects were selected among patients of a previous study that comprises familiar and sporadic ET cases, whereas control individuals were recruited in the Movement Disorders Unit of the Donostia University Hospital (in San Sebastian, Spain). A new informed consent was signed by every participant in our study, which was approved by the ethics committee of the Donostia University Hospital [25,26]. For each individual, an electrophysiological test (EPT) and fMRI are available. In the test, individuals draw a line, the Archimedes’ spiral, and do some handwriting both with the dominant and the non-dominant hand [25,26]. In this study, we use only the spiral of Archimedes (Figure 2). The database presents variability regarding the amplitude, frequency, and pattern of the tremor; scale of diagnosis; and demographic data (gender and age). As far as the individuals selected for the study are concerned, a subset of samples of the spiral of Archimedes is picked up from the original database analyzing genetic and clinical assessment. With regard to the samples of the spirals studied, these samples (or spiral drawings) come from subjects diagnosed with ET according to clinical and electrophysiological criteria [27]. Tremor rating scales provide crude, non-linear and subjective assessments of the severity of tremor. Among the most commonly used scales, the Fahn-Tolosa-Marín tremor rating scale (FTM) uses a grading of 0–4 to evaluate tremor in Archimedes’ spiral drawings. The scale of Bain and Findley uses a grading of 0–10. Both scales have a strong logarithmic relationship with the amplitude of the tremor measured by means of a digital tablet (consistent with the Weber-Fechner law). Actually, the digitizing tablets are much more accurate than the clinical classifications, but this advantage is mitigated by the natural variability of the tremor, and hence, this is why the study that we present makes sense [28]. Within the group of individuals with ET, only the samples of the hand affected by essential tremor are used, but five patients are not taken into consideration due to the poor quality of their samples. For the control individuals, the best sample is selected (mostly the dominant hand), and for some individuals the non-dominant hand is also used. Therefore, this sub-database BIODARWO is made of 51 samples selected from the original data set: 27 samples selected from the control individuals, and 24 samples (the hand affected by tremor) from the ET individuals. A higher database would be very useful. Unfortunately, as often happens, the study has a limited number of samples. This is a classical Biomedical Engineering problem. In the bibliography, we can find many real examples in the same scenario. One of the typical cases is the modelling and exploring of the genes that caused a specific type of cancer. In this case, there are more than 4000 genes-features, so a few of them are selected to be used as features to feed a classifier. Additionally, under Hospitals’ Ethical Committees’ advisors, this is a standard study, and working with other subsets of the data in a previous phase, we already obtained results using a smaller sample size, but also a smaller set of features, reaching similar numerical accuracies. The complexity of the problem lies in the difficulty in obtaining fMRI samples in these patients that can only be gathered as part of a research study, not in habitual clinical practice. For this reason, the sample is limited. The study can be considered a feasibility study, which is not the same as a pilot study [29].

## 3. Methodology

### 3.1. Derived Measures

**Figure 3:**
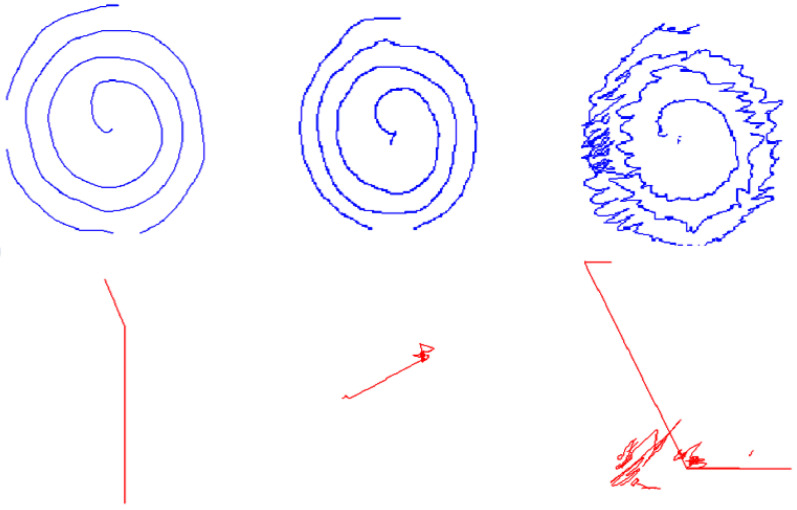
Sketching of the spiral of Archimedes carried out by (from left to right): a CR individual, an ET individual at an early stage, and an ET individual at an advanced stage. Both pen-down (top image in blue) and pen-up (bottom image in red) are shown simultaneously.

**Figure 4:**
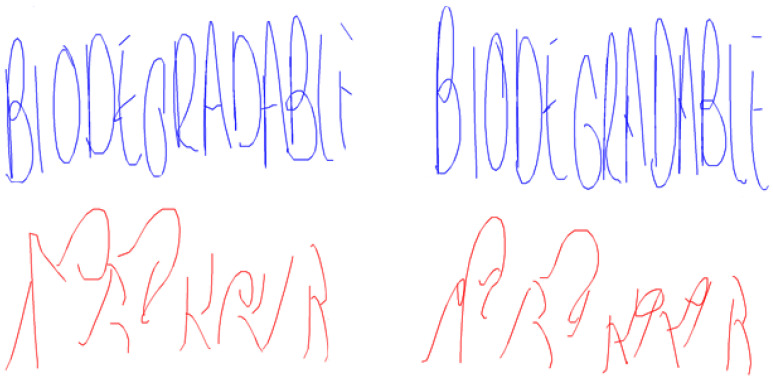
Pen-down (**top images**) and pen-up (**bottom images**) trajectories of different captures of biodegradable for the control group.

Thanks to on-line drawing it is possible to evaluate the time spent and the pressure exerted while writing, from a quantitative point of view. For instance, it is well known that AD patients spend more time and produce simpler and softer strokes [12], while people with ET and Parkinson’s Disease produce less stable pressure patterns [12,30,31]. In [29], the ability of the individuals is related to pressure and in-air trajectories (their length) [32,33,34,35]. Another new interesting characteristic of modern on-line handwriting analysis is that it can bear in mind information collected while the pen does not exert pressure on the surface. Figure 3 represents the drawing of the spiral of Archimedes carried out by three individuals: a control individual, an ET individual at an early stage, and an ET individual at an advanced stage [26]. Pen-down (on-surface trajectories) are shown in blue, while pen-up (in-air trajectories) are shown in red. Figure 4 represents the writing of the word BIODEGRADABLE from the CR group to better illustrate the pen-up and pen-down information. The in-air information, or pen-up, is shown in red, and the on-surface information or pen-down is shown in blue.

Therefore, the movements of the hand when drawing or writing some text can be divided into two categories: on-surface trajectories (pen-down), and in-air trajectories (pen-up) [35]. The pen-down corresponds to the movements of the writing device when it touches the writing surface. Each of these on-surface trajectories is a visible stroke. The pen-up corresponds to the movements of the hand when changing from one stroke to the next, that is, there is no pressure at all on the writing surface. Our past studies on recognition of people based on biometry showed that these two kinds of data are complementary [10], and their discrimination capability is similar, even when using a database of 370 individuals [11,34].

### 3.2. Extraction of Features

In this study, we present a preliminary experiment whose main goal is to establish thresholds for some handwriting related biomarkers. As mentioned above, it belongs to a broader research study aimed at early diagnosis of ET. Therefore, the search of features in our study aims at preclinical evaluation orientated towards the definition of effective tests for diagnosing ET [9,12,20,25,26].

#### 3.2.1. Linear Features

**Figure 5:**
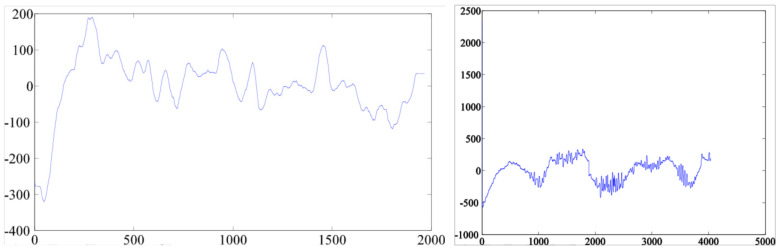
Projections of the polar components of the drawing of the spiral of Archimedes carried out by a control person (**left image**) and a patient with ET (**right image**).

**Figure 6:**
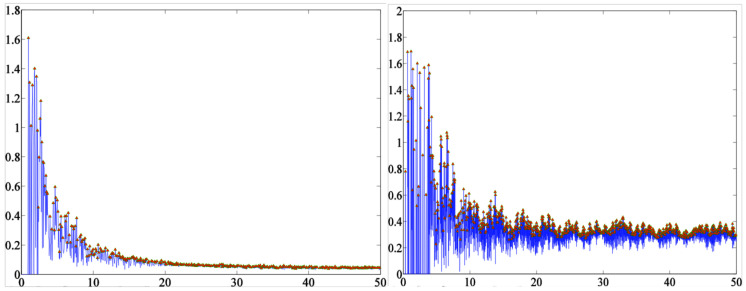
Spectrum of the coordinate X of the pen-down signal for a individual of the control group (**left image**) and an individual with ET (**right image**).

The aim is to automatically differentiate between ET individuals and healthy people from the analysis of several linear features (LF), which are attributes or variables linearly dependent on data, and variants (min, max, median, and mean), extracted from their handwritings:

1. Time-related measures: time on-surface, time in-air, and total time (on-surface plus in-air times).
2. Spatial components and variants: coordinates X and Y, altitude angle O, azimuth angle A, angle Z and modulus R polar components and their projections for both in-air and on-surface signals (Figure 5 shows, in the drawing of the individual with ET, an illustration of the distortion of the polar components).
3. Pressure and variants (min, max, mean, median, std).
4. Features of dynamic nature and variants: speed and acceleration for both in-air and on-surface signals.
5. Zero crossing rate.
6. Frequency related features: spectral components for both in-air and on-surface signals (Figure 6) [26].

#### 3.2.2. Non-Linear Features

The objective is to use the analysis of non-linear features (NLF) extracted from the subjects’ samples of handwriting in order to distinguish automatically between healthy individuals and ET sufferers. The NLFs under consideration are: fractal dimensions (FD) and their variants (min, max, mean and median) for the pressure and the spatial components x, y, computed using the Higuchi algorithm (HFD) and the Castiglioni algorithm (CFD); the entropy (E) and its variants (min, max, mean and median) for the pressure and the spatial components x, y; and signal distortion by analyzing local peaks.

The fractal dimension of an object is a measure of its self-similarity. A self-similar object is approximately similar to a part of itself, that is, if we examine it closer, we will find smaller versions of it. In order to quantify self-similarity, we can measure the number of building-blocks that are part of a pattern; this measure is the so-called fractal dimension (FD). For a given waveform, there is no reference of the FD value it should have. Additionally, as signals are not usually stationary, in most techniques, short signal sections are used in order to calculate features from the waveform. For example, the signal can be split into short pieces in order to calculate the features of each piece, which is the technique that we have taken into account. In other words, we compute the fractal dimension of short segments of the signal, and observe the evolution of the results along the whole signal, trying to discover fractal characteristics that could be helpful to identify different elements of that signal. There are several algorithms that can be used to measure the FD. In our study, the focus is on the options especially suitable for the analysis of time series which do not need any previous modeling of the system. Some of these methods include the Higuchi algorithm [36], the Katz algorithm [37] and the Castiglioni algorithm [38]. Higuchi and Castiglioni were selected, as in previous works in under-resourced conditions, this approach has proven to be more accurate [7,8,39,40,41].

On the other hand, physical systems entropy is a disorder measure. For a single discrete random variable *X*, the entropy *H* measures its average uncertainty. Shannon entropy [42] is computed as:

(1) $$H\left(X\right)=-\underset{x_{i}}{\sum }p(x_{i})logp\left(x_{i}\right)$$

### 3.3. Sets of Features

Next, we present the sets of features used in the experimentation:

1. Set of Linear Features (LF), this set will be described in 3.3.1.
2. Set of Non-Linear Features (NLF), which consists of LF and the set that will be described in 3.3.2. (Higuchi (HFD), Castiglioni (CFD), entropy (E), and fractal dimension (F)): LFHFD, LFCFD, LFE, LFHFDE and LFCFDE.
3. The resulting set of features after selection by means of ANOVA: SLF (Selection of Linear Features), SLFHFD, SLFCFD, SLFE, SLFHFDE and SLFCFDE.

Table 1 shows the complete list of acronyms and their meanings.

### 3.4. Automatic Selection of Features

The automatic feature selection is based on statistical tests (ANOVA). The MatLab one-way balanced analysis of variance function (anova1) is used [43].

Two or more columns of data that represent independent samples that contain mutually independent observations are compared with regard to their means. The test computes the probability (*p*-value) under the null hypothesis that every sample is drawn from populations with the same mean. If p is near to zero, we can say that the null hypothesis is very unlikely, so we can assume that the mean of at least one of the samples is significantly different from the means of the other samples. Commonly used significance levels are 0.05 or 0.01; in our case it is 0.05. The standard ANOVA test differentiates the variability of the data into two types: on the one hand, the variability due to the discrepancy between the sample means, or variability between groups; and on the other hand, the variability due to the differences between the data in each sample and the sample mean, or variability inside the groups. The size of the F-statistic and the *p*-value are derived from the box plot of the samples. Significant differences between the center lines of the boxes lead to large values of F, and therefore small values of *p*.

### 3.5. Automatic Classification

The principal aim of this study is the selection of potentially useful handwriting features useful for preclinical evaluation to define tests in order to diagnose ET. Such features should differentiate between the group of control individuals (CR) and the group of individuals with essential tremor (ET). As these techniques have to be useful in real environments for real-time applications, a second objective is to optimize the computational cost. Therefore, the model of automatic classification will be performed bearing in mind these two goals. Three different classifiers implemented in the WEKA software suite [44] were used. All configurations were oriented to real time:

1. A Support Vector Machine (SVM) using polynomial kernel (e = 1.0).
2. A Multi Layer Perceptron (MLP) using neuron number in the (unique) hidden layer (NNHL) = max (Attribute/Number + Classes/Number) and training step (TS) = NNHL × 10.
3. K-Nearest Neighbor Algorithm or k-NN (using *k* = 1).

In order to evaluate the results, we used Accuracy (Acc in %), Classification Error Rate (CER in %) and Accumulative Classification Error Rate (ACCER in %) for every model [5,24]. In the steps of training and validation we used k-fold cross-validation (*k* = 10). Cross-validation is a robust technique for the selection of variables [45]. Thanks to repeated cross-validation (as computed by WEKA), robust statistical tests are possible. We used the measurement provided by the function “Coverage of cases” of WEKA (confidence interval at 95% level) as well.

## 4. Results and Discussion

The experiments were performed using the balanced subset BIODARWO. The aim of the experiment was to assess the potential of selected features to automatically measure the degradation of the Archimedes’ spiral drawn by subjects that suffer ET. Therefore, the previously defined sets of features have been assessed to differentiate properly between control and essential tremor groups.

### Extraction of Linear and Non-Linear Features

**Figure 7:**
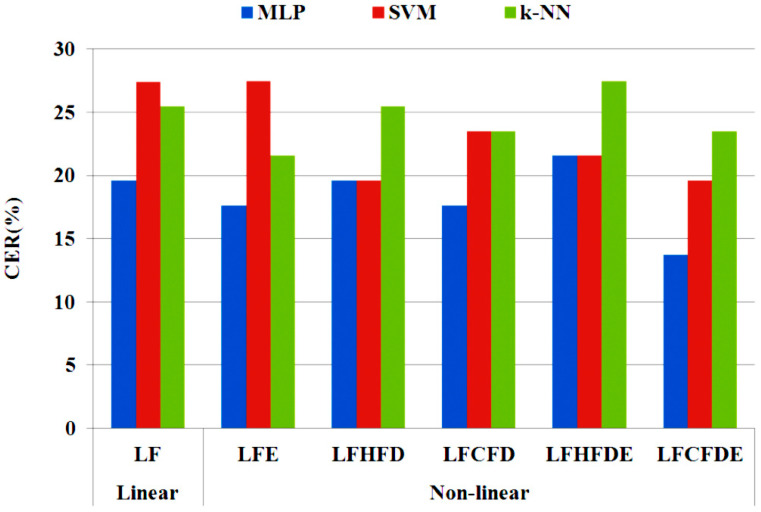
CER (%) for the three classifiers considered with linear and non-linear sets of features.

In a first phase, linear and non-linear features were extracted using the techniques of Section 3.4 (see Table 2), and an automatic classification was performed by means of the classifiers enumerated in Section 3.5. Figure 7 summarizes the resulting CERs (%) for the different classifiers, with linear and non-linear feature sets. In these results we can observe that:

- The sets of non-linear features provide better results for every classifier.
- MLP and SVM have the best results in most of the cases.
- Both FD features are able to provide better results for every classifier.
- Shannon entropy has the best performance for MLP when is combined with CFD.
- The best option is LFCFDE with Castiglioni FD, entropy and MLP, where the CER is 13.73% with 225 features.

**Figure 8:**
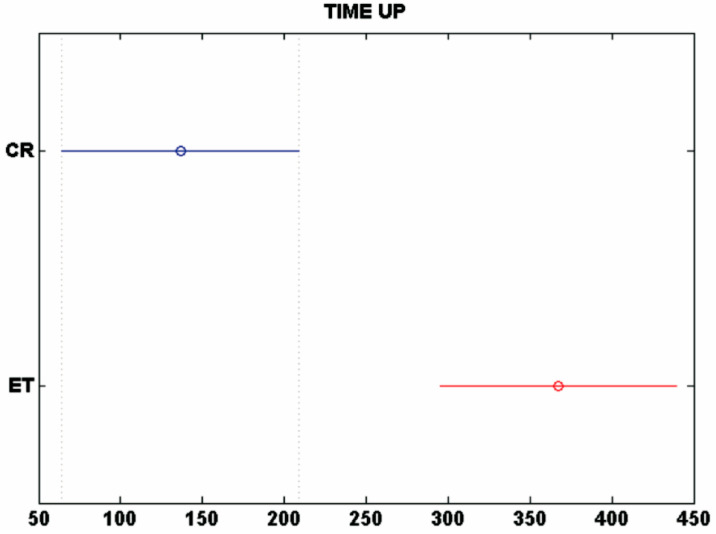
Example of ANOVA analysis: time-up for the CR group in blue and the ET group in red.

**Figure 9:**
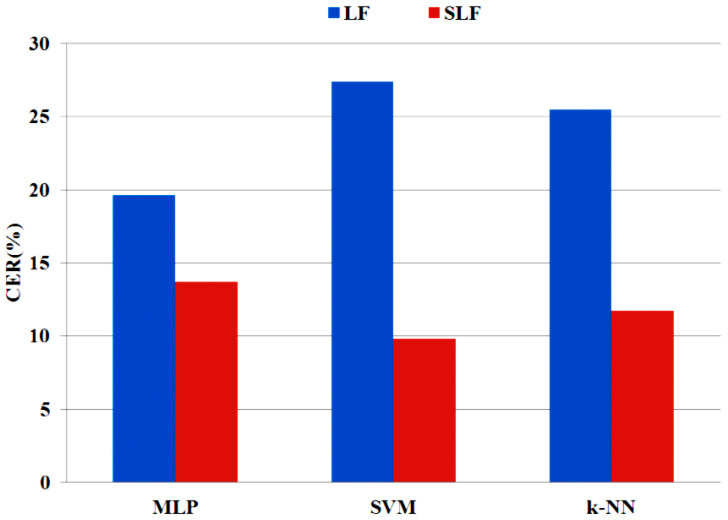
CER (%) yielded by the classification algorithms for the entire set of linear features (LF) and the subset of selected linear features (SLF).

**Figure 10:**
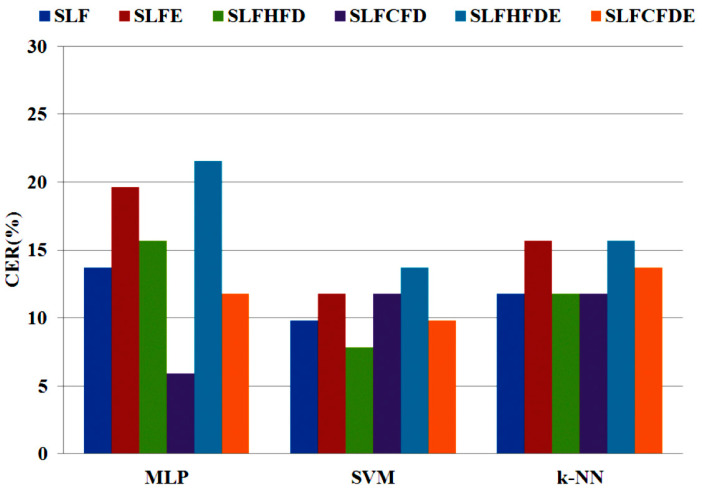
CER (%) yielded by the classification algorithms for the reference SLF and the selected sets of non-linear features.

In a second stage, an automatic selection of features is performed by applying the ANOVA test described in Section 3.5. Figure 8 shows the details for the selected feature time-up (in-air), which represents the time that the pen is not on the writing surface. In Table 2, we can see that the number of features (NF) for linear and non-linear cases is reduced by about 65% after the selection process. Finally, an automatic classification process was applied to the entire database using the previously described classification algorithms. Figure 9 and Figure 10 graphically summarize the CERs (%) yielded by each algorithm when considering linear and non-linear sets of features. In these results, we can observe that:

- The automatic selection of features improves the results for most of the algorithms when linear features are considered (Figure 9). With the selected features, MLP is the algorithm that performs worst.
- When the non-linear selected features are included, the results are better in most cases with regard to the LFs.
- Both non-linear features (FD and entropy) improve the results regardless of the applied classification algorithm.
- Shannon entropy performs better with SVM, and when combined with FD.
- The best option with regard to feature sets is SLFCFD with Castiglioni FD and MLP with NF = 77 features (CER = 5.89%). The same feature set provides the best results for SVM when applying the Higuchi algorithm for FD.
- Good results are also obtained with *k*-NN, requiring a lower computational cost with the sets that include feature selection by ANOVA and FD.

**Figure 11:**
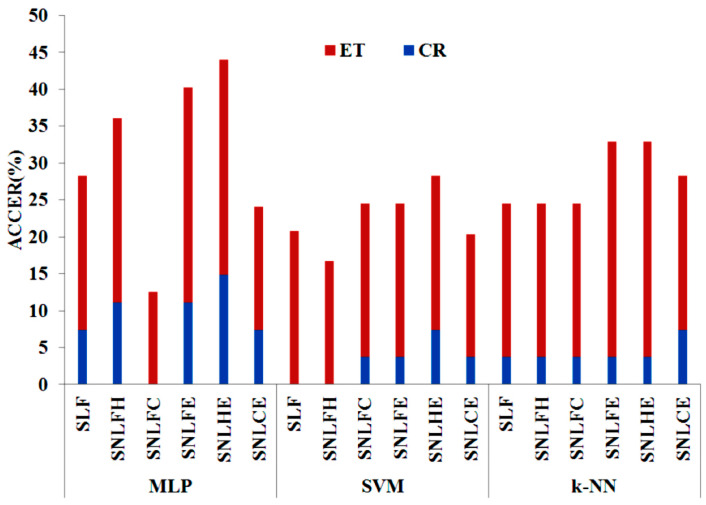
Accumulative Classification Error Rate (%) for the three tests defined and each set of features. ET = Essential Tremor, CR = control group.

Accumulative Classification Error Rate (ACCER in %) for CR and ET groups is shown in Figure 11. A lower accumulative error rate is achieved in the experiments based on the MLP classifier that includes non-linear features. Experimentation based on the SVM classifier with non-linear features (CFD and entropy) shows better performance with regard to both groups (CR and ET).

**Figure 12:**
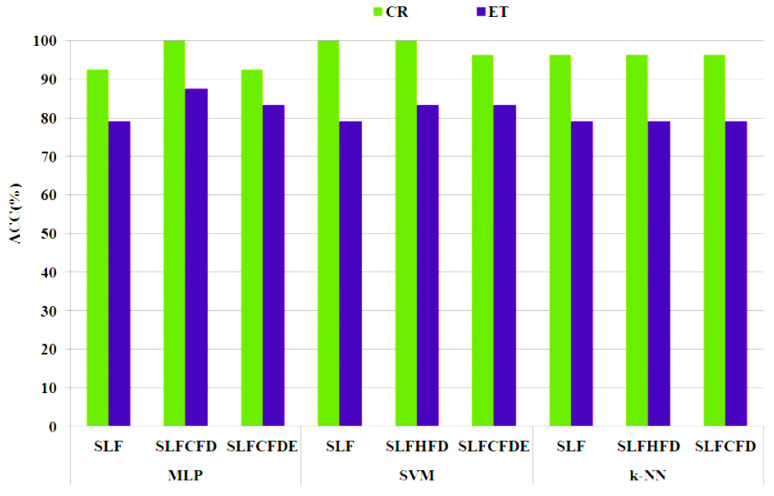
Accuracy (%) for each group (CR and ET) and classification algorithm when considering the best performing sets of features.

Figure 12 shows, for each class (ET and CR), a detailed analysis of the accuracy (Acc in %) of the results yielded by the three classification algorithms with the reference SLF and the two best options for the non-linear selected features. Please note that:

- Most of the selected non-linear feature sets outperform the original ones. *k*-NN with a lower computational cost yields good results when using the selected features and FD.
- The sets of features that contain the features selected by the ANOVA test plus non-linear features are the best option with regard to optimum and balanced performance for both CR and ET groups.
- SLFCFD with MLP is the best option with regard to computational cost, balanced results of groups and the number of features with an accuracy of 94.11% with 77 features (Table 3).

The results reported in this paper are similar to other results with data from the same database, already published, and using other features/classifiers. We are developing this project in collaboration with Physicians and according to them this can be of real help for them. Our models fulfil the medical requirements. We also have to take into account that the final model will integrate not only information about the Archimedes’ spiral, but other information provided by specialists as well. Concerning the CER, which is related to the error still present, is not null, because in some of the cases we were not able to detect the tremor, or it is mixed with other types of tremor due to different causes in controls. On the other hand, this system provides a useful tool for drug monitoring, which is another field of interest for physicians, because it can help to adjust the amount of medication taking into consideration how the tremor changes because of the administered drug.

## 5. Conclusions

The principal goal of this work is the analysis of features in the drawing of the Archimedes’ spiral, the gold standard test for ET diagnosis. The selection of non-linear biomarkers from handwriting and drawings belongs to a larger cross study aimed at diagnosing essential tremor. We have analyzed classic linear features (static and dynamics) and non-linear features (fractal dimension and entropy) as well. The analysis took into consideration several Machine Learning algorithms, and the automatic selection of promising features by means of the ANOVA test. We achieved optimal results even after reducing the number of features about 65% using the selection process. Furthermore, according to the health specialists working in the wider cross study, the obtained biomarkers are suitable and can be applied to the early diagnosis of ET. Therefore, we plan to integrate these new biomarkers with the formers derived in the study of the Biodonostia Health Institute. Finally, we must emphasize that the use of these methods could clearly benefit the development of low cost, more sustainable, high quality, and non-invasive methods readily adaptable to users and their environments. From a social and economic approach, they can be very valuable and beneficial in real complex environments. In future research lines, other non-linear features and automatic selection of algorithms could be explored.

## Figures and Tables

**Table 1:** Complete list of acronyms and their meanings.

Acronym	Description
**ET**	Essential Tremor
**PD**	Parkinson’s disease
**FD**	Fractal Dimension
**HFD**	Higuchi Fractal Dimension
**CFD**	Castiglioni Fractal Dimension
**E**	Shannon’s Entropy
**LF**	Linear Features
**NLF**	Non-linear Features
**LFXFD**	Linear Features and Fractal Dimension
**LFXFDE**	Linear Features and Fractal Dimension and Shannon’s Entropy
**SLF**	Selection of Linear Features
**SLFXFD**	Selection of Linear Features and Fractal Dimension
**SLFXFDE**	Selection of Linear Features and Fractal Dimension and Shannon’s Entropy
**SVM**	Support Vector Machine
**MLP**	Multi Layer Perceptron
**NNHL**	Neuron number in the hidden layer
***k-*NN**	*K*-Nearest Neighbor
**NF**	Number of Features
**TS**	Training Step
**CER**	Classification Error Rate
**Acc**	Accuracy
**ACCER**	Accumulative Classification Error Rate

**Table 2:** Sets of features and number of features (NF).

	LF	LFHFD	LFCFD	LFE	LFHFDE	LFCFDE	SLF	SLFHFD	SLFCFD	SLFE	SLFHFDE	SLFCFDE
NF	186	213	213	198	225	225	70	73	77	76	79	86

**Table 3:** CER (%) yielded by each classification algorithm with linear features (LF), selection of linear features (SLF), and the best set of selected features plus FD (computed using the Castiglioni algorithm) and entropy (SLFCFDE).

	MLP	SVM	*k*-NN
**LF**	19.61	27.41	25.5
**SLF**	13.73	9.81	11.77
**SLFCFD**	11.77	9.81	13.73
